# Supplementary material for: Pre-Clinical Evaluation of Tenofovir and Tenofovir Alafenamide for HIV-1 Pre-Exposure Prophylaxis in Foreskin Tissue
Source: Pharmaceutics. 2022 Jun 16;14(6):1285. doi: 10.3390/pharmaceutics14061285 (PMC9227286; doi:10.3390/pharmaceutics14061285)
Supplement: Supplementary file 1 [file pharmaceutics-14-01285-s001.zip › pharmaceutics-1733055-supplementary/pharmaceutics-1733055-supplementary/Supplementary files/Figure S1.pdf]

(a) (b)

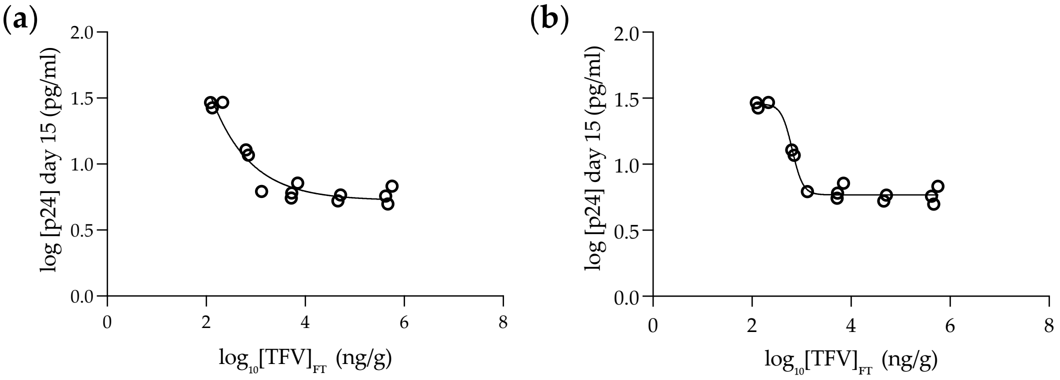

(c)

Model	r^2	Probability (%)	AICc
Linear	0.6430	0.01354	-41.58
One-phase decay	0.9251	99.99	-59.39
4-parameter model	0.9796	> 99.99	-72.56

Figure S1. Non-linear model of TFV dose and p24 response correlation. (**a**) Exponential decay fit and (**b**) 4-parameter non-linear model for tissue explant concentrations of TFV and p24 levels in culture supernatant 15 days post-ex vivo challenge with LVT (○). Data are mean (SEM) from three independent experiments performed in triplicates. FT, foreskin tissue. (**c**) Comparison of linear with non-linear models. r^2 = (1-) the sum of the squared distances from each fitted curved divided by the squared distances from a horizontal line. AIC = Akaike information criterion value. The 4-parameter non-linear model provided lower AIC values indicating a better fit than an alternative one-phase decay model. One-phase decay model: Y=(Y0 - Plateau)*exp(-K* X) + Plateau. Sigmoidal 4-parameter model: Y=Bottom + (Top-Bottom)/(1+10^((LogEC50-X)*HillSlope)).
