# Supplementary material for: Pre-Clinical Evaluation of Tenofovir and Tenofovir Alafenamide for HIV-1 Pre-Exposure Prophylaxis in Foreskin Tissue
Source: Pharmaceutics. 2022 Jun 16;14(6):1285. doi: 10.3390/pharmaceutics14061285 (PMC9227286; doi:10.3390/pharmaceutics14061285)
Supplement: Supplementary file 1 [file pharmaceutics-14-01285-s001.zip › pharmaceutics-1733055-supplementary/pharmaceutics-1733055-supplementary/Supplementary files/Table S1.pdf]

Table S1. Tenofovir concentrations after *ex vivo* dosing of foreskin explants with tenofovir alafenamide (TAF) and tenofovir (TFV).

		TFV							
		Foreskin tissue[a]		Wash Solution[c]		Culture Supernatant[b]		Total[d]	
Drug	Dose (µg/mL)	ng/gram	% <LLQ	ng/mL	% Loss	ng/mL	% Loss	ng/mL	% Loss
**TFV**	5000	483,169 (42,391.7)	0	35,078 (4,160.0)	7.26	450,164 (63,543.5)	93.2	485,243 (67,497.8)	100.4
	500	48,768 (3,102.0)	0	3555 (232.5)	7.29	50,098 (5,040.8)	102.7	53,652 (5,179.3)	110.0
	50	5,868 (572.5)	0	436.0 (70.1)	7.43	4,866 (326.7)	82.9	5,302.7 (390.7)	90.4
	5	885.7 (215.0)	0	50.9 (2.1)	5.74	535.7 (63.7)	60.5	586.3 (65.5)	66.2
	0.5	156.3 (29.0)	0	9.3 (2.3)	5.93	78.0 (19.4)	49.9	87.0 (21.7)	55.8
**TAF**	15	12,453 (2,299.6)	0	63.6 (8.2)	0.51	1,060 (115.9)	8.51	1,123 (116.3)	9.02
	1.5	1,267 (275.0)	0	8.78 (1.0)	0.69	183.0 (12.8)	14.4	192.0 (13.0)	15.1
	0.15	803.7 (614.2)	0	1.84 (0.3)	0.23	22.7 (10.7)	2.79	24.3 (10.7)	3.02
	0.015	63.7 (24.6)	0	2.35 (1.1)	3.67	25.0 (14.6)	39.0	26.7 (14.4)	41.4
	0.0015	68.7 (42.7)	0	2.59 (1.8)	3.78	16.0 (8.5)	23.5	18.3 (10.3)	27.2

Data are expressed as the mean (SEM).

[a] n=1 replicate analysed for each donor per dosing level.

[b] n=1 for each donor per dosing level.

[c] 3 x wash solutions pooled (~1800µL), n=1 per dosing level.

[d] TFV concentrations in the dosing supernatant (harvested after a 2hr incubation period in the presence of foreskin tissue) were not determined, as concentrations were outside of the assay calibration range.
