# Supplementary material for: Pre-Clinical Evaluation of Tenofovir and Tenofovir Alafenamide for HIV-1 Pre-Exposure Prophylaxis in Foreskin Tissue
Source: Pharmaceutics. 2022 Jun 16;14(6):1285. doi: 10.3390/pharmaceutics14061285 (PMC9227286; doi:10.3390/pharmaceutics14061285)
Supplement: Supplementary file 1 [file pharmaceutics-14-01285-s001.zip › pharmaceutics-1733055-supplementary/pharmaceutics-1733055-supplementary/Supplementary files/Table S2.pdf]

Table S2. Tenofovir-DP concentrations after *ex vivo* dosing of foreskin explants with tenofovir alafenamide (TAF) and tenofovir (TFV).

		TFV-DP									
	Dose	Foreskin tissue[a]		Dosing supernatant[b]		Wash Solution[c]		Culture Supernatant[b]		Total	
Drug	(μg/mL)	pmol/gram	% <LLQ	pmol/mL	% Loss	pmol/mL	% Loss	pmol/mL	% Loss	pmol/mL	% Loss
**TFV**	5000	1,917 (1,088.6)[d]	0	82.9 (52.0)	4.32	214.0 (185.6)	11.2	234.4 (52.7)	12.2	531.3 (290.3)	27.7
	500	1,307 (236.8)	0	24.1 (11.4)	1.84	21.2 (3.5)	1.62	17.9 (1.3)	1.37	63.1 (16.3)	4.83
	50	120.3 (51.5)	0	4.34 (0.96)	3.60	2.62 (0.33)	2.17	7.11 (0.0)	5.91	7.9 (3.9)	11.7
	5	40.7 (21.5)	67	<LLD	N/M	1.73 (0.95)	4.26	<LLD	N/M	1.7 (0.9)	4.26
	0.5	10.8 (3.7)	100	<LLD	N/M	1.43 (0.65)	13.2	<LLD	N/M	1.4 (0.6)	13.2
**TAF**	15	675.0 (184.1)	0	15.3 (0.0)	2.26	7.66 (1.3)	1.13	14.5 (0.0)	2.14	17.6 (8.6)	5.54
	1.5	111.0 (27.8)	17	<LLD	N/M	2.10 (0.0)	1.89	<LLD	N/M	2.1 (0.0)	1.89
	0.15	57.0 (7.3)	67	<LLD	N/M	<LLD	N/M	<LLD	N/M	N/M	N/M
	0.015	16.1 (14.1)	75	<LLD	N/M	<LLD	N/M	<LLD	N/M	N/M	N/M
	0.0015	21.5 (11.7)	83	<LLD	N/M	<LLD	N/M	<LLD	N/M	N/M	N/M

Data are mean (SEM) of three independent experiments performed in triplicates.

[a] n=2 replicates analysed for each donor per dosing level.

[b] n=1 for each donor per dosing level.

[c] 3 x wash solutions pooled (~1800μL), n=1 per dosing level.

[d] replicates from one donor (TFV dose = 5000 μg/mL) excluded due to significant (>200%) TFV-DP loss in the wash and culture media.

N/M: Not measurable.
